# Post-disaster mobilities of Muslim typhoon survivors: How gendered religious preferences and discrimination shape socio-spatial exclusions in Catholic-majority Cagayan de Oro, Philippines

**DOI:** 10.1177/23996544231200002

**Published:** 2023-09-30

**Authors:** Christine Gibb

**Affiliations:** 6363 University of Ottawa, Canada

**Keywords:** Mobility, exclusion, religion, Philippines, disaster, gender

## Abstract

Natural hazards don’t care who you worship. However, the evacuation camps, transitional housing sites and relocation sites aimed at helping disaster survivors do. Empirically, this paper explains a puzzle in which Muslim survivors of Typhoon Sendong in the Philippines were all but absent in official post-disaster spaces of this Catholic-majority country. Based on qualitative interviews, focus groups and site visits, I identify two exclusionary mechanisms: (1) prejudices, preferences and practicalities, and (2) socio-spatial design of official post-disaster spaces. This paper argues that by studying Muslim survivors’ post-disaster mobilities, we see that discrimination along the lines of religion, as it plays out in everyday gendered religious socio-spatial practices, repels survivors from accessing evacuation camps and other post-disaster spaces. This is important for two related reasons. One, these humanitarian spaces claim to be inclusive yet, in practice, deter would-be migrants on the basis of religion. Two, religiously-informed gender relations shape the politics of disaster recovery processes, which further exacerbate inequities post-disaster.

## Introduction: Religion in the aftermath of disasters

Typhoon Sendong, as it is known locally in the Philippines, made two things clear: natural hazards don’t care who you worship - or if you even worship at all - but the official evacuation camps, transitional housing sites and relocation housing sites of state and humanitarian actors do. On 16 and 17 December 2011 Tropical Storm Sendong (international name: Washi) hit the city of Cagayan de Oro (CDO), Philippines, catalysing unprecedented levels of death, displacement and destruction (LGU of CDO, 2012; NEDA, 2012; OCD, 2012). According to the Local Government Unit, the flooding displaced 228,576 people (38,071 families), but floodwaters did not discriminate between informal and formal settlements, poor and affluent neighbourhoods, makeshift and concrete houses (LGU of CDO, 2012: 8). Of the displaced, 65,046 (13,321 families) spent one or more nights in an evacuation camp (LGU of CDO, 2012: 8). Fewer moved into transitional housing sites and relocation sites. The reasons why CDO survivors avoided official post-disaster spaces are common; for example, survivors had access to alternative free lodging from family or close social networks or could stay at their worksite, they had financial resources to pay for hotel rooms or rental apartments, or they were reluctant to leave their homes due to attachment to place or fear of theft. Additionally, in CDO, religion emerged as a critical factor shaping gendered post-disaster mobilities among Muslim survivors, indicative of the persistent intertwining of social and spatial relations in a disaster context.

State and humanitarian actors who design and run evacuation camps, transitional housing sites and relocation sites, or what I call ‘official post-disaster spaces’, herald their adherence to humanitarian principles, namely impartiality, humanity, neutrality and independence (Collinson and Elhawary, 2012; Grombach Wagner, 2005; Hilhorst and Jansen, 2010). The actors overseeing post-disaster housing similarly claimed their spaces observed humanitarian principles. Thus, official post-disaster spaces were assumed to meet all survivors’ basic needs, and to welcome all people regardless of religious affiliation. Yet these same actors noted that in most cases ‘Muslims just don’t go to evacuation camps or temporary and permanent housing.’ This observation sets the empirical puzzle the paper tackles: why weren’t Muslim survivors in official post-disaster spaces?

Answering the question is urgent. With increasing frequency and intensity of natural hazards-cum-disasters linked to climate change (IPCC, 2014), environmental and disaster mobilities are set to become more important around the world. The uneven impacts of disasters raise critical questions around political and social justice, as recognized by the emergent focus in disaster management on disaster justice^1^ (Lukasiewicz, 2020). Truly inclusive humanitarian post-disaster spaces will thus be needed. Yet the role of religion in disaster response, experiences, and mobilities remains understudied (Feener et al., 2015; Gaillard and Texier, 2010).

To investigate the empirical puzzle, I study the post-disaster mobilities of CDO’s Muslim survivors. I argue that discrimination along the lines of religion, as it shapes everyday gendered socio-spatial realities and the embodied experiences of religious beliefs and practices, systematically excludes survivors from accessing post-disaster spaces. The following section reviews the reasons for uneven post-disaster mobilities, highlighting the curious dearth of research focused on religion. This literature review opens the possibility of situating religion as a demonstration of post-disaster agency. Next, I draw extensively on the CDO case, analysing why Muslim survivors avoided official post-disaster spaces, and how religiously-informed gender relations shaped disaster recovery processes. Following the approach of Aijazi and Panjwani (2015: 28), on their excellent study on ‘religion in spaces of social disruption’ in post-landslide Pakistan, I focus on survivors’ everyday socio-spatial experiences of religion. I pay particular attention to the gendered elements, finding two exclusionary mechanisms: (1) prejudices, preferences and practicalities, and (2) the socio-spatial design of official post-disaster spaces. These mechanisms underscore that religion matters in shaping gendered disaster mobilities.

## Disaster mobilities, and the exercise of agency through religion

Post-disaster, the capacity to exercise mobility is highly unequal (Cook and Butz, 2016; Sheller, 2014, 2016a; Tuitjer, 2019). Many factors explain such differentiated access. Curiously, the potential of religion in shaping post-disaster mobilities remains underexplored in both the mobilities and the disasters literature. Although some disaster literature engages with religion, most key sociological, anthropological and geographic disaster texts do not, or do so in a limited way (Feener et al., 2015; Aijazi and Panjwani, 2015). This brief review points to key areas to meaningfully insert religion. This section ends with an overview of the historical context of the social-spatial marginalization of CDO’s minority Muslim population. It is warranted because disasters (Bankoff, 2001; Hewitt, 1983; Pelling, 2001; Pelling and Dill, 2010; Wisner et al., 2004), like mobilities (Cook and Butz, 2016; Sheller, 2016a; Tuitjer, 2019), are best understood situated within their unfolding geopolitical histories.

### Disaster mobilities

Natural hazards, and the disasters they precipitate, reshape mobilities– not only for people, but also for financial aid, relief goods, and disaster and rebuilding discourses (Cook and Butz, 2016; Sheller 2014, 2016a, 2016b; Tuitjer, 2019; Zickgraf, 2019). These mobilities can make the difference between life and death, and influence relief, recovery and rebuilding processes. Early environmental migration literature spotlighted the double dilemma of trapped populations: people with little capital cannot afford to move away from environmentally risky areas, yet the same lack of capital diminishes their capacity to cope and adapt to environmental shocks and change, effectively trapping them in areas susceptible to environmental change (McLeman, 2013; U.K. Government Office for Science (GOS), 2011). Scholars emphasized distinguishing between *trapped* populations who cannot leave and *immobile* populations who chose to stay, although the line separating these categories is blurry (GOS, 2011). In this early literature, immobility is oversimplified as trapped populations lacking financial capital (GOS, 2011). More recent scholarship challenges the immobile-trapped binary, and insists upon placing immobility on a mobility continuum in which agency can permeate (Ayeb-Karlsson, 2020; Schewel, 2020; Zickgraf, 2019).

This paper uses a mobilities entry point to study mobilities linked to disasters because it most closely explains observed movements. In addition to long-distance and long-term movement, mobilities studies examines the short-distance, short-term movements (or lack thereof) of people, as well as those of objects, ideas and capital in physical, digital and blended spaces (Cook and Butz, 2016; Sheller, 2014, 2016a, 2016b; Tuitjer, 2019). Mobility studies’ emphasis on *relational* dynamics accentuates the political and ethical aspects of uneven mobility in which power, exclusion and mobilities intersect (Ayeb-Karlsson, 2020; Cook and Butz, 2016; Sheller, 2014, 2016a). This focus recalls Doreen Massey’s (1994: 149) insistence of framing mobility in a power geometry, where ‘different social groups, and different individuals, are placed in very distinct ways in relation to these flows [of technology, capital, communication, etc.] and interconnections.’ Consequently, as Massey (1994: 149) famously wrote, mobility is never equally accessible to everyone, ‘some people are more in charge of it than others; some initiate flows and movement, others don’t; some are more on the receiving-end of it than others; some are effectively imprisoned by it.’ A mobilities framing also fits with the fluidity and range of mobility pathways and experiences of individuals and groups over time and space (Zickgraf, 2019); it recognizes that immobility is never absolute and is produced through the interplay of ‘internal constraints,’ ‘retain factors’ and ‘repel factors’ (Schewel, 2020: 339).

Natural hazards catalyse differentiated mobilities for specific social groups whereby only certain people can move (GOS, 2011; McLeman, 2013), or are permitted to move (Cook and Butz, 2016; Tuitjer, 2019), or feel their individual mobility is supported by gendered social norms and power relations (Ayeb-Karlsson, 2020; Ayeb-Karlsson et al., 2019). Humanitarian workers and politicians, for example, flew in and out of disaster sites in Pakistan and Haiti on private aircraft, while local residents were trapped *in-situ* until damaged infrastructure was repaired (Cook and Butz, 2016; Sheller, 2016b). After the 2011 flooding in Bangkok, Thailand, a combination of factors, notably the legal status of asylum seekers and refugees, the risks posed by exposing themselves to state authorities in official post-disaster spaces, and pejorative discourses around racialized migrant workers, instigated the creation of new temporary mobility practices among non-Thai survivors distinct from their Thai survivor counterparts (Tuitjer, 2019). In three coastal cyclone affected sites in Bangladesh, gendered disaster immobility was linked not only to financial or physical hurdles, but also to ‘social values and reasoning, … gender identity… psychological trauma and feelings, [and] … the gendered division of knowledge and decision-making power’ (Ayeb-Karlsson, 2020: 9). These examples demonstrate that myriad social markers affect survivor security, mobility, and access to disaster relief. These markers include ethnicity, race, nationality, cultural heritage, citizenship status, legal status, class, gender, age, physical ability, and sexuality (Ayeb-Karlsson, 2020; Bradshaw, 2015; Gaillard et al., 2008; Hyndman, 2008; Juran, 2012; Sheller, 2016a; Tuitjer, 2019; Wisner et al., 2004). Curiously, most disaster scholars have not studied how religion shapes mobility.

### Religion and post-disaster agency

This gap is peculiar, given that religion shapes social reality, especially in the aftermath of a disaster when pre-existing inequalities are amplified (Fothergill, 1998; Hewitt, 1983; Pelling and Dill, 2006, 2010; Wisner et al., 2004). The salience of religion in development is now widely recognized, correcting an earlier gap (Deneulin and Zampini-Davies, 2017), but still remains understudied with relation to environmental mobilities or disasters. (The 2010 special issue of *Religion* and the 2015 special issue of *The International Journal of Mass Emergencies and Disasters* are notable exceptions.) The following paragraphs briefly define religion, as I use it in this paper, and outline how religion is understood to impact post-disaster processes. Following Aijazi and Panjwani (2015), this review argues for situating religion, as it shapes everyday socio-spatial realities and embodied experiences of religious beliefs and practices, as a demonstration of post-disaster agency.

In their review of 30 years of development studies and religion, Deneulin and Rakodi (2011) argue that religion is heterogeneous and dynamic, and that it articulates particular norms for evaluating development processes. They define religion ‘as an institutionalized belief system that unites a community of believers around social practices’ (Deneulin and Rakodi, 2011: 47). Accordingly, in this paper, the term ‘religion’ does not refer exclusively to organized religion as it is prescribed in particular religious texts like the Qur’an or the Bible. Rather, I mean it as a cultural interpretation of a formal religion, specifically Islam and Christianity, as it is lived out in the everyday private and public lives of individuals, and in the policies and practices of faith-based organizations (FBOs). Religion can thus be treated as everyday experience, as it is embodied in gendered socio-spatial practices.

Religion permeates myriad aspects of disasters. Pre-disaster, religious beliefs inform hazard risk perceptions and adherence to early warning systems (Ayeb-Karlsson, 2020; Ayeb-Karlsson et al., 2019). In a survey of 2386 adolescents in tsunami-susceptible coastal areas in Indonesia, Hall et al. (2022: 7, 8) found that both religion and gender directly influenced risk perception; ‘Muslim participants were associated with higher earthquake and tsunami risk perceptions compared to Catholic, Protest, Hindu and other religious participants’ and young women participants were ‘more likely to report believing they were susceptible to a tsunami, [and] less likely to feel that they could save themselves if a tsunami occurred.’ After a disaster, religious discourses can spur individual or collective responses, expedite or slow recovery processes, help survivors comprehend their experiences or access specific cultural tools for recovery, confer the vocabulary for anchoring survivors in their specific social worlds while communicating with the outside, and underpin everyday acts of resistance that challenge dominant disaster relief narratives (Aijazi and Panjwani, 2015; Bankoff, 2001; Feener et al., 2015; Gaillard and Texier, 2010; Gaillard et al., 2008; Nguyen, 2019; O’Connell et al., 2017; Sohrabizadeh et al., 2018). As such, religion is important for coping and networking on an individual or household level. The effects of religiosity, and especially how religiosity is employed as a coping method, can differ between disaster-affected men and women (Sohrabizadeh et al., 2018). On an institutional level, FBOs and formal religious bodies play diverse roles, notably collecting monies, disbursing aid, mobilizing volunteers, sponsoring relocation housing, navigating bureaucracies, and financing *in-situ* rebuilding (Aijazi and Panjwani, 2015; Bulmer and Hansford, 2009; Feener et al., 2015; McLaughlin, 2018; Reale, 2018). Most studies about religion and disasters, however, concentrate on instrumental aspects instead of critically scrutinizing the roles, motivations and impacts of these FBOs, echoing the trend in the religion and development literature more broadly (Clarke, 2007; Deneulin and Zampini-Davies, 2017; Jones and Petersen, 2011; Tomalin, 2012). In contrast to approaches focused on the religious interventions of FBOs or faith leaders in driving disaster management (McLaughlin, 2018), or on the religious struggles of survivors post-disaster (O’Connell et al., 2017), Aijazi and Panjwani (2015: 33) attend to ‘religion as an encompassing field through which the lives of communities moved and unfolded as they engaged in their journeys to [disaster] recovery.’ This everyday religion approach foregrounds religion in post-disaster spaces, turning them into spaces into sites of agency. Human agency, Kuus (2019: 163) reminds us, is ‘the capacity to act in a given context,’ and thus demands a focus on the specificity of agency. This approach promises rich insights:

By paying attention to spaces and scales previously deemed private or apolitical, the conception of who qualifies as a political agent is broadened, and new ways to rethink agency, structure, and action are created. …This broadening of agency is open to fresh considerations of emotion, affect, and religion in individual and collective action. It takes any person’s or group’s capacity to act as ambiguous and contingent: not progressive or reactionary, enabling or disabling as such, but always a process that is rooted in specific social contexts and needs to be situated in these contexts. (Kuus, 2019: 165)

Using a similar approach to agency, Aijazi and Panjwani (2015) studied everyday religion in disaster recovery in Khyber Pakhtun Khwa province following the 2010 Pakistan Monsoon Floods. They observed numerous examples of what they called political acts that helped ‘to redefine the political to include micro spaces of subversion within disaster relief: communities resist grand humanitarian narratives and poor program design by communicating in a language that feigns compliance with relief actors while preserving their dignity’ (Aijazi and Panjwani, 2015: 45). Survivors’ political acts included: attributing the assistance they received to Allah and not to relief agents, prioritizing the construction of purdah walls around their homes, and avoiding official displacement camps run by government or private relief agencies.

Such acts do not enact a heroic agency. Rather, they echo Mahmood’s (2001, 2005 in Aijazi and Panjwani (2015): 43) articulation of agency as not just the capacity for progressive change, but also the capacity to endure, suffer and persist. Such a framing of agency disputes the optimistic discourses of agency, empowerment and self-sufficiency that characterize country-level disaster resilience programs and international frameworks to reduce disaster risk (Grove, 2014). Human agency should not presuppose action (Schewel, 2020), or an intent for betterment (Kuus, 2019). Framing agency in this way broadens the conceptualization of religion beyond a means for coping or for implementing a more effective and efficient disaster recovery. The mobilities of CDO’s Muslim survivors of Typhoon Sendong illustrate this assertion.

### The CDO context: Pre-Sendong socio-spatial religious divisions

Historically, religion has conditioned social and spatial development of the Philippines. The political, educational, judicial systems and other social institutions that developed first under colonial rule, and later in an independent republic, were largely conceived and run by a Christian elite (Moreno, 2006; Tan, 2009). Even today, in a secular state, institutions continue to reflect a Christian worldview. For centuries, these institutions created social and spatial distinctions among the archipelago’s inhabitants. In particular, they marginalized the minority Muslim population (Abinales, 2000, 2010). In CDO, the coexistence of communities separated along religious lines persisted after Typhoon Sendong. The usual habit of the city’s Christian majority to not interfere with the city’s Muslim population went uninterrupted.

In the early 2010s CDO’s population was predominantly Christian. About 10 percent of city residents were Muslim; most had arrived in the 1990s or later. Muslims shared many city spaces with non-Muslim city residents, especially commercial spaces. The spatial distribution of the city’s Muslim population and Islamic institutions was much more confined than their non-Muslim counterparts. Most Muslims resided in five of the city’s eighty *barangays.*^2^ Fewer than 10 mosques served the Muslim population. Unlike many of the Christian churches, no mosque was situated in the city center; instead, mosques were tucked away in a maze of buildings or in peripheral *barangays*. Islam’s imprint on the city was much less visible than that of Christianity, belying claims of religious inclusivity.

Officially, CDO welcomed everyone, even calling itself the ‘City of Golden Friendship.’ It celebrated the history of Muslims in Mindanao in an exhibit at a university museum. Public spaces, such as markets, shopping malls, schools, restaurants, and businesses were open to, and operated and frequented by, people of all faiths. Yet, signs of Islam in public spaces were much less conspicuous than signs of Christianity. Few businesses in CDO advertised clever wordplays and symbols inspired by Islam. A handful of *jeepneys* and *motorelas* were notable exceptions.^3^ Several small shops at the main public market, and at a major shopping mall catered specifically to a Muslim clientele, for example by selling Muslim women’s clothing. Other shops were owned and operated by Muslim families selling goods with broad appeal, such as mobile phones and prepaid credit, jewellery, CDs and DVDs, apparel and accessories, and beautiful *malongs.*^4^ Apart from the *malongs*, the only clues to the religious affiliation of the shopkeepers were banter in the Maranao language and the hijabs and chadors worn by some women owners and clerks. Private opinions did not match the official discourse of religious inclusivity. In conversation, some non-Muslim residents expressed their personal ignorance and misgivings about Muslims – always a generic Muslim. The remarks of a middle-aged Christian businessman are illustrative: ‘the Christian Filipinos at [site] won’t say anything to Muslims who break the dress code because they are afraid of what might happen to them. There’s a real fear of Muslims among Christian Filipinos. Were a Muslim to walk into the restaurant, all the Christian Filipinos would be afraid.’ Consequently, while not officially barred from any city space, Muslim residents typically did not match their non-Muslim counterparts in their use of and control over urban space. These personal prejudices and the underlying historical Christian hegemony limited Muslim residents’ access to urban spaces.

Politically, CDO’s Muslim population wielded little clout. Even the *barangay* with the largest Muslim population had no Muslim *barangay* councillor. Similarly, few or no Muslims were elected to City Council, or were employed in municipal, provincial and regional government offices.^5^ Muslim residents could voice concerns but were not guaranteed an empathetic audience. For example, an official from a *barangay* with a large Muslim population told me there were no Muslims there. We were a five-minute walk away from the Sharief Alawi Islamic Center where I had just held a focus group with Muslim residents. The Sharief Alawi Islamic Center boasted the largest mosque in Northern Mindanao and CDO’s only *madrasah* (Arabic school) (Sharief Alawi Islamic Center, no date). Yet, it was not served by regular *jeepney*, *multicab* or *motorela* routes. The lack of public transportation to important Muslim spaces indicates widespread inattention to the socio-spatial practices of the city’s Muslim population. It reflects wider religious prejudices that ultimately restricted Muslim survivors’ mobility and access to humanitarian and state-sponsored resources post-Sendong. The subordinate position of the city’s Muslim population meant that their post-disaster mobility and treatment were heavily influenced by historical relations, contemporary prejudices and interactions between Muslims and non-Muslims.

## Unravelling an empirical puzzle: Why can’t Muslim survivors access official post-disaster spaces?

### Methodological approach

This research is part of a larger project on the post-disaster experiences of Typhoon Sendong survivors in Cagayan de Oro, Philippines. The project was approved by the Research Ethics Board at the Université de Montréal (CERFAS-2012–13-071-D). The following findings are based on 10 months of ethnographic accompanied fieldwork in the Philippines in 2010, 2012 and 2013 (see Gibb, 2021 on accompanied fieldwork). The full set of empirical material consists of interviews, focus group discussions, participatory videos, non-participant observation, counter-mapping activities, and document analysis. The analysis here is based upon a conventional content analysis of 107 interviews with key informants representing religious groups, government agencies, local and international NGOs and humanitarian groups, private companies and educators involved in post-disaster efforts and the reports they produced. The official claims made by these sources were then contrasted with the experiences of survivors, as expressed in 79 survivor interviews with adults (56 women, 23 men), focus group discussions, and participatory videos. Survivors had varied social and economic backgrounds. Many were urban poor and worked in the informal economy. Some resided in evacuation camps and transitional housing sites, while others did not. Most were Christian, reflective of the city’s Catholic-majority population. Many of the methods utilized were mobile research methods insofar as they were literally done ‘“one the move” and “simulate[d] intermittent mobility”’ as participants retraced their post-disaster trajectories during their interactions with the research team (Sheller, 2014: 801). Scholars studying environmental mobilities have successfully used such mobile qualitative methods (Tuitjer, 2019). A methodological approach to ‘zero in on the granular specificity of agents’ and the ‘sensory feel of everyday political practice’ captures the subtle exercise of agency (Kuus, 2019: 167).

### Sampling and target population

Despite being affected by Sendong, I was repeatedly informed that ‘no Muslim died in Sendong’ and ‘there are no Muslims in evacuation camps.’ Accordingly, I purposively selected Muslim survivor interviewees and focus group participants to unpack these statements. I used convenience and snowball sampling to connect with Muslim participants who worked as shop vendors or owners in the main public market, or as employees at the regional office of the National Commission on Muslim Filipinos (NCMF-X), or who lived in a *barangay* with a significant Muslim population. It is these narratives around which the paper revolves. Research activities were often conducted in three languages, with Muslim participants speaking mostly in Maranao, an educated – usually younger – relative translating between Maranao and Visayan, and my research assistant translating between Visayan and English. I am neither Muslim, nor Filipina. These aspects of my positionality facilitated conversations about what was characteristic of Muslims and what was characteristic of Filipinos in the aftermath of Sendong. When women participants learned that I was a new mother, they often discussed their experiences relationally as mothers, sisters, daughters, wives.

According to NCMF-X records, 1460 Muslim households were affected by Sendong in CDO, including 407 households in one *barangay* alone. Yet, the Muslim community was conspicuously absent from official post-disaster spaces. Instead of accessing official post-disaster spaces, Muslim survivors sought refuge in the houses of local relatives and friends or in their mosque, and availed of financial and material assistance from Muslim compatriots, similar to Muslim survivors in other disasters (Aijazi and Panjwani, 2015). As a senior NMCF official explained, ‘relief assistance was given in the mosques. That’s where people went first. Muslim evacuees go to (1) mosques, (2) *barangay* centers, (3) evacuation centers, in that order.’ Only a handful of Muslim individuals – never families – went to evacuation camps and received relocation housing. However, these individuals left. Few Muslim survivors felt safe in these spaces; they had preferable options. This result is hardly surprising given that disasters can expose, reproduce and exacerbate inequalities (Fothergill, 1998; Hewitt, 1983; Pelling and Dill, 2006, 2010; Wisner et al., 2004), and CDO’s Muslim community was already marginalized.

Muslim survivors avoided official post-disaster spaces, for reasons closely tied to religion. Muslim survivors cited religion-based discrimination, beliefs and cultural expectations as major factors. While these elements operate in everyday city life, there is sufficient space for them to mostly remain in the background. In the cramped quarters of official post-disaster spaces, these elements rise to the fore, with gendered impacts. The following pages consider how and why Muslim survivors absented official post-disaster spaces. My evidence is interpreted in two exclusionary mechanisms that, together, created uneven mobility. The mechanisms emerged through a close reading of the data, not pre-established categories. The mechanisms are (1) prejudices, preferences and practicalities, and (2) the socio-spatial design of official post-disaster spaces. For each mechanism, I first present the evidence, and then discuss how the evidence shows that religion affects gendered post-disaster mobilities. Although each mechanism is presented separately, in practice, they are interlinked.

### Prejudices, preferences and practicalities

Given that disasters lay bare existing inequalities, it is unsurprising that parallel but non-mixing trajectories persisted post-disaster with Muslim survivors’ post-disaster trajectories largely avoiding the Christian survivors’ post-disaster pathways. This section considers factors shaping the post-disaster mobilities of Muslim survivors that underscore the salience of gender relations and ideologies, of faith and kin-based networks, and how discrimination on the basis of religion is tied up in them. The following examples show that religion matters to post-disaster mobilities and the gendering of these pathways.

Prevailing discourses about Muslims served as one mechanism of exclusion. Like the endeavours of other experts or trustees (Li, 2007), the design of the disaster response and of official post-disaster spaces was not underlain with insidious intentions. Yet, presumptions of Muslim survivors’ insularity, capacity and preference to take care of themselves, as well as ignorance or partial knowledge of particular religious needs, resulted in exclusion. For example, the tendency of Muslim survivors to avoid the government- and church-run evacuation camps in CDO was noted by officials, and explained away as a family-size phenomenon. In answering my question on why Muslims avoided evacuation camps, a government official in charge of evaluating disaster losses and allocating disaster relief and relocation housing responded using stereotypes, ‘because maybe they have plenty of relatives,’ and not data collected by her office. An employee of the archdiocese attributed the avoidance to culture, noting that ‘most avoided the evacuation centers because that’s their culture.’ Another key informant explained his organization’s disaster rebuilding focus in a specific geographical area because ‘people [here] are Christian. [Our staff] know how to work with them versus with the people living in the [site name], … whose catchment area is in a Muslim area.’ That these types of assumptions were held by the people in charge of post-disaster housing had repercussions for the design of housing, who is targeted, and which sites are resourced.

Muslim survivors preferred drawing upon Muslim networks. Muslim households sought out and enacted post-disaster mobilities distinct from state- and humanitarian-sponsored pathways that channelled survivors from evacuation camp to transitional housing site to permanent relocation site. Whenever possible Muslim survivors remained in their own homes, ‘even though’ explained one young woman, ‘it is really dirty and muddy and stinky.’ As illustrated by the following focus group excerpts, Muslim participants justified their aversion of evacuation camps based upon pre-existing religious-based socio-spatial divisions and animosity.

‘The Muslim doesn’t mingle with Christians because of cultural barriers. We mingle with our relatives and other Muslims, so we [Muslims and Christians] don’t mingle in recovery.’

‘Muslims themselves avoid the Christians. They [Muslims] feel animosity and want to avoid chaos.’

‘Most [Muslim] Sendong victims don’t like to live with Christians. And other Christians don’t want Muslim neighbours. Some Christians expressed their dislike [of Muslims] and that they don’t get along well [with Muslims].’

Participants also justified their avoidance based on religious needs and cultural expectations around hospitality.

‘Muslims would choose to stay with relatives who understand their religious needs.’

‘Muslims must always be near a mosque because the men need to go to the mosque to pray five times per day.’

‘If a Muslim sees a fellow Muslim who is affected by a calamity, they will feel obligated to help them (even if they are not related by blood). Muslims are very hospitable people. It is also a Filipino thing to be hospitable.’

In addition to feeling more welcomed by those with a shared faith, CDO’s Muslim survivors stressed the continuity of religious-based socio-spatial divisions post-disaster. Moreover, these survivors *could* avoid evacuation camps by utilizing kin and religious networks. As an NCMF-X employee recounted, ‘Muslim people preferred to go to their relatives in [*barangays* not affected by Sendong]. Most Muslims have relatives living in CDO. Muslims and Filipinos are hospitable, are caring. So if you don’t have relatives, other Muslims will invite you to stay with them. You are not expected to pay rent to them at this time.’ These sentiments (and economic considerations) were reflected in post-disaster mobilities; if Muslim survivors could not stay in their own houses, they typically stayed with relatives in CDO or further afield in another city, and occasionally with other non-relative Muslims or in one of the city’s mosques. Aijazi and Panjwani (2015) observed similar preferences in Pakistan where Muslim survivors opted to stay with family, or at the mosque or *madrasah*. The avoidance by Muslim survivors of evacuation camps rendered them mostly ineligible for the free state-, church- and humanitarian-sponsored relocation housing, which required a minimum stay at an evacuation camp, thereby further entrenching CDO’s pre-existing religious-based socio-spatial divisions.

The intertwining of gendered economic and religious considerations also shaped the mobilities of Muslim survivors in the weeks immediately after Typhoon Sendong. While Muslim women were economically active household members and recounted how Sendong disrupted their livelihood endeavours, remaining close to work sites was particularly important for men. This priority resulted in differing degrees of geographic, social and economic mobilities among Muslim men, women and children. Muslim families frequently adopted a temporary split householding strategy: the husband remained in the family’s CDO house while the wife brought the children to stay with relatives in Marawi City, 100 km southwest of CDO, until water and electricity were restored and ‘things were better.’ The man was expected to continue his paid work, often involving small-scale entrepreneurial activities at, or near, the city’s main market, or river sand quarrying from the Cagayan River. This prioritizing of livelihood continuity is not unique to CDO’s Muslim survivors; it is consistent with the findings of Sohrabizadeh et al. (2018) who found that, in Iran, Muslim men disaster survivors prioritized resolving post-disaster livelihood challenges over religious concerns. In CDO, Muslim men’s livelihoods were strongly rooted in specific locales. It was deemed safer, more comfortable and more appropriate for the women and children to leave the city and live with extended family members while their CDO homes were cleaned and repaired. Most Muslim split householding families eventually reunited in CDO. Only a few houses of Muslim Sendong survivors were permanently deserted by their former inhabitants.

The split householding pathway demonstrates a highly gendered migration pattern based on a gendered division of labour and entrenched constructs of gender underpinned by a specific understanding of religious teachings. That is, Muslim women survivors frequently noted that, according to the Qur’an, men dictate women’s mobility, and where they go or not. As such, respecting the wishes of male family members was deemed appropriate, respectful and pious. A quote from a Muslim woman vendor is illustrative, ‘Muslim women strongly believe what is written in the Qu’ran. Once a place is full of sin, the people are punished. Ideally Muslim women don’t wander - they need their husband’s permission to go places.’ As a woman NCMF employee recounted, adherence to religious teachings was strengthened by the fact that ‘No Muslim died [in CDO]. The faith saved us. No Muslims died because of Allah - there was trauma, property damage, etc. but no deaths.’ Curiously, no Muslim men survivors drew upon religious ideology to explain mobility decisions. Many Muslim survivor households had the financial means and familial networks to facilitate post-disaster mobilities beyond the official trajectories. These assets make it possible for households to prioritize religious considerations in determining mobilities. That these survivors forged their own paths is indicative of the discrimination they experienced along religious lines, the importance of everyday religious socio-spatial practices, and the agency they drew upon post-disaster.

The very real fear of discrimination based on religion justified avoiding official post-disaster spaces. Prejudices against CDO’s minority Muslim population persist, even though they do not escalate into violence as in other parts of Mindanao. Despite the good intentions of officials to create inclusive post-disaster spaces, Muslim survivors feared ostracization. Muslim survivors contended they would be misunderstood by Christians, with whom they would necessarily share space. This was especially problematic if, as sometimes happened, two families were forced to inhabit a single tent. As one informant put it, ‘if your tentmates are Christian they will misunderstand you.’ Distinguishing among CDO’s Muslim and non-Muslim survivors was relatively easy. As in other post-disaster situations (Fothergill, 1998; Reyes and Lu, 2016), it was mostly the women who lined up for relief and store vouchers. Many, but not all, of CDO’s Muslim women wear a veil outside their homes. As one interviewee explained, ‘the people distributing relief won’t tell you that you won’t receive the gift cheques because you are Muslim. But, if they see you wearing the veil, they’ll just tell you there are no coupons left.’ As such, a visible marker of social location became a tool for denying disaster relief. Despite this repudiation, Muslim women survivors persisted with their social-spatial veiling practices, refusing to hide their Muslim identities and to forgo their everyday embodied religious practices in exchange for disaster relief.

Muslim women participants recounted incidents demonstrating discrimination. In one instance, after registering as official Sendong survivors, several women discovered they had been subsequently removed from the *barangay* list and were thus ineligible for government cash assistance. No explanation was offered to justify their removal. Moreover, the NCMF’s survivor list was neither recognized nor used by the key government agencies allocating housing. Many Muslim survivors were thus deprived of legitimate survivor status, and the opportunity to relocate as documented survivors.

In a focus group with Muslim men and women survivors, participants described how Muslim women survivors were excluded from receiving relief intended for Sendong survivors. The topic of yellow cards (i.e., government-issued survivor cards) elicited lots of animated and loud responses. The participants recounted that it would be announced that a regional government agency would distribute aid on a particular date, so the women would go with their yellow cards at the appointed date, only to find out that their names were not on the list. Two women explained:

‘This is because, at the time, [government agency] sent volunteers to check on the flooded areas, to assess damages and to assign help. There was no careful checking. If you are ‘just flooded’ you get nothing. If you are ‘washed out’ you get something. [When the agency] checked in their computer they said that they [Muslim survivors] were only flooded, so they received nothing from the government.’

‘They say that other people in [subdivision of barangay] got cash assistance. But, some people were removed from the [beneficiary] list. I do not know why.’

Other tactics similarly deprived Muslim survivors from obtaining relief goods and gift certificates redeemable at local supermarkets. From their houses the women would see large army trucks pass along the main road carrying relief goods, then stop at the *barangay* hall. The women would go to the *barangay* hall to ask when the relief goods would be distributed. They would be given a time, usually later that day or the next day, but when they returned at the appointed time, there would be nothing left. Other times the relief items were substituted with inferior quality items; women recalled one particular delivery when the original relief packages consisting of 5 kg bags of good quality rice and cans of halal beef were replaced with 1 kg bags of government rice (considered by many Filipinos to be of poor quality) and canned sardines. Other times, officials would explain that the relief packages contained pork that Muslims would not eat, even though the women could see non-pork items. The perception of discrimination in relief distribution is not unique to CDO’s Muslim women survivors. Similar accounts were described by survivors in other areas, as well as by local NGOs working on behalf of survivors both Muslim and Christian. It is an experience shared by women survivors of other disasters, for example, in the Eastern Visayas (Nguyen, 2019), Metro Manila (Reyes and Lu, 2016) and Bangladesh (Alam and Rahman, 2014).

Claims of intentional discrimination were tempered by additional comments from focus group participants and by government and Catholic Church officials. Several Muslim survivors explained that families were able to return to their homes with support from the government and the archdiocese. As one senior NCMF official noted, ‘Several Muslims were not helped by relief aid. This is because of a characteristic of Muslims - they would rather go to relatives and friends for help than to Christians. That’s why we have a Mindanao problem.’ In discussing the distribution of relief goods and services, financial and in-kind support for cleaning and repairing houses, and relocation housing, all key informants insisted that the rules were followed and there was no preferential treatment. They claimed it was a question of eligibility, not religious affiliation. Yet, as I argue in the following section, the *in*attention to religious preferences in the socio-spatial design of official post-disaster spaces produced equally exclusionary impacts.

### The socio-spatial design of official post-disaster spaces

The physical and social design and the geographical location of evacuation camps, transitional housing sites, and permanent relocation sites dissuaded Muslim survivors from staying in official post-disaster spaces. Although observance of gender-based religious obligations yielded the same outcome for men and women, the impetus for the post-disaster mobilities of each group differed. This section articulates how religious norms about veiling and the need for truly *private* private spaces dissuaded Muslim women from accessing official post-disaster spaces, while religious norms about daily prayer inside a mosque and the need to access a particular public space deterred Muslim men. These physical design and geographical siting deterrents were further reinforced by the social design of official post-disaster spaces.

#### Physical design: The need for truly private private spaces post-disaster

**Figure 1.**
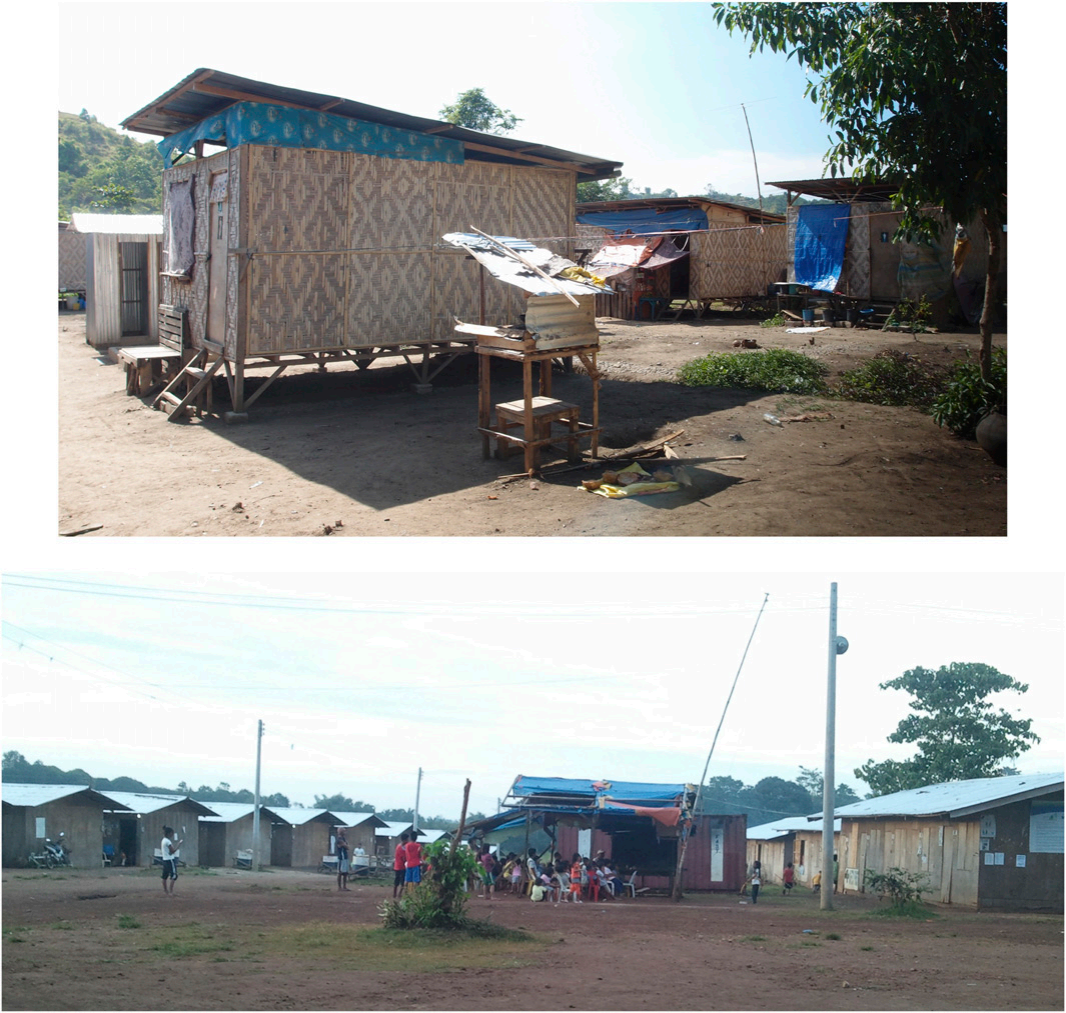
Top – *Amakan* housing at one of CDO’s transitional sites (6 April 2013). Bottom – Wooden bunkhouses with concrete foundations at a transitional housing site (14 March 2013). No site includes a purdah wall. Photos by author.

In their recovery, as in their pre-disaster lives, Muslim women interviewees prioritized gender-specific religious norms in their use of space. Officials overlooked these norms in designing CDO’s post-disaster spaces, despite genuine efforts to create safe spaces for women. This situation is not unique to CDO; for example, among tsunami-affected women in Tamil Nadu in India, ‘the privacy, security, sanitation and health needs of women were not factored into the design and construction’ of temporary shelters (Juran, 2012: 16; see Ayeb-Karlsson, 2020 for similar observations in Bangladesh). Official post-disaster spaces were simultaneously private spaces insofar as they were intended to be residences for typhoon-affected households, and public spaces insofar as they were cramped quarters with little actual privacy. Extant public buildings and public grounds were rapidly repurposed as evacuation camps with large open areas subdivided with blankets into dozens of individual family units (Gibb, 2022). Transitional housing units consisted of emergency tents from humanitarian organizations laid out in a grid, of temporary structures made of locally-sourced materials with *amakan*^6^ walls and corrugated metal or *nipa*^7^ roofs, and of wooden bunkhouses^8^ with cement foundations (Figure 1). That the purportedly safe spaces lacked privacy had important repercussions on Muslim women survivors, and their households in that they opted to avoid these spaces. Although CDO’s Muslim survivors could not change the design of official post-disaster spaces, they exercised agency through their deliberate avoidance of them.

The socio-spatial practice of veiling emerged as a critical issue for Muslim women survivors – but not their male counterparts – in explaining how the delicate balancing act of recovering from the disaster while embodying their religious beliefs shaped post-disaster trajectories. Muslim women wear the veil for a variety of reasons (Marchand, 2009; Mohanty, 1988; Secor, 2002). In her book *Politics of Piety,* Saba Mahmood argues the veil should be understood as (1) an expression of the identity of Muslim women or (2) both a means to achieve piety, modesty, and other virtues and an end indicating the achievement of these attributes (Landry, 2010). For Muslim women survivors in CDO, the veil primarily represented the latter option. The lack of privacy in an evacuation camp rendered it a public space with no truly private spaces. As a focus group participant noted, ‘it’s not easy for Muslim women to mix with men, but in the evacuation center, you have no choice but to mix with men.’ Consequently, as other focus group participants explained, for faith-based reasons, a Muslim woman would have to be covered all the time because she cannot reveal her hair to men other than her husband or close relatives. In other words, religious beliefs that require minimal mixing between men and women deterred Muslim women from seeking out official evacuation camps.

The spatiality of the socio-spatial practice of veiling became evident over the course of a three-hour group discussion and mobile interviews with Muslim survivors. The discussion took place in the *madrasah*, in the presence of several men, including an Imam. There, all the women wore veils. After leaving *madrasah*, we walked along the residential streets, passing by the *barangay* hall, a *motorela* station and several *sari-sari* shops. One woman removed her veil during the tour. When we entered into private homes, with only women present, almost all removed their veils. The fact that different women wore and removed their veils in different spaces shows that socio-spatial practices of veiling are the exercise of an individual woman’s agency and not her adherence to blanket rules imposed by the state or other authority. It shows, too, that veiling is linked to personal choice (agency), which can reflect different rationales for wearing the veil. Thus, embodied, rather than imposed, religious norms about veiling and the need for truly private spaces dissuaded Muslim women from accessing evacuation camps. As such, both their embodied religious beliefs and practices, and their agency in enacting them, shaped their post-disaster trajectories.

Veiling is a socio-spatial practice that affects women’s mobility through urban spaces. As Secor (2002: 8) explains, the city has ‘different, spatially realized sets of hegemonic rules and norms regarding women’s veiling …[which] should be understood to differ in terms of formality, enforcement, stability, and contestation.’ It is not just the rules, but how individual women interpret and apply these gendered rules to their own lives and mobility practices. Gender-based restrictions render adaptation to co-ed post-disaster spaces harder for Muslim women than Muslim men. Adhering to these rules was one reason why the few Muslim survivors who ventured into CDO’s temporary housing sites were single men and not Muslim women or Muslim families. Here, religious norms affected gender-based access to and exclusion from official post-disaster spaces. Enacting these norms demonstrates agency, even though this exercise of agency resulted in Muslim women and their households losing out on disaster aid.

#### Geographical siting: The need to access public religious spaces post-disaster

Geographical proximity to a mosque was a priority for Muslim men and a deterrent to relocating temporarily or permanently. A young woman vendor and her mother explained the gendered expectations for praying at a mosque.

Daughter: Muslims pray five times a day. My husband goes to the mosque to pray in [barangay name]. I only go to the mosque two times per year. Women typically stay home for their praying or just pray at their work. Every Sunday [some] Muslim women go to the seminar area next to the mosque to read and understand the Qur’an and Islamic ways.

Mother: It is not obligatory for a woman to go to the mosque every day, but it is obligatory for men to go every day. I don’t have a set schedule for attending the mosque. Sometimes I go up to four times a month.

Not surprisingly, most of CDO’s Muslims are clustered in *barangays* where residences and worksites are within walking distance of a mosque. Everyday gendered mobilities thus shaped and were shaped by everyday religious practices and subjective understandings of religiosity. Gendered mobilities did not just disappear with the disaster; instead, the gendered prerequisites (and agency in enacting these needs) of Muslim men shaped the post-disaster mobilities of entire households.

The siting of official post-disaster spaces posed significant problems. The city’s 10 mosques were far from the temporary and permanent relocation housing. Not only were these sites geographically distant from mosques but they were also inaccessible via lack of public transportation, or public transportation that was expensive, long and uncomfortable. This situation rendered it difficult for Muslim men to practice their faith in a formal religious building. For example, would-be mobile Muslim survivors could not just attend another mosque near a hypothetical new home. In contrast, many migrant Christian survivors began attending religious services at another church when they were moved to a new evacuation, transitional or permanent relocation housing site. Hundreds of churches were dispersed throughout the city, so finding a new place of worship was relatively easy for Christians. Relocated Christian survivors frequently explained that they began attending services at a new church – even if it was a different denomination – because it was closer. Having to worship in another church did not strongly influence the post-disaster mobilities of Christian survivors. No such option existed for Muslim survivors who did not want to relinquish their faith. The difficulty of getting to public religious spaces to carry out everyday religious practices thwarted Muslim men – and by extension their households – from seeking relocation housing. Thus, a commitment to socio-spatial religious practices compelled Muslim survivors to forge post-disaster trajectories that eschewed official post-disaster spaces.

#### Social design: Imposing Christian values in post-disaster spaces

It was more than just the physical design and siting of official post-disaster spaces that neglected religious considerations. It was also their social design. Donors and officials in charge of post-disaster housing embedded exclusions on the basis of religion into the institutional design of relocation camps. Religious criteria strongly deterred potential residents from seeking access, thereby propelling alternative post-disaster mobilities on the basis of religious identity. To support this claim, the following paragraphs examine the values training program that would-be residents of the Xavier Ecoville relocation site must complete before moving into a relocation home.

Xavier Ecoville is unique among CDO relocation sites, and even among relocation sites in the entire archipelago. Unlike other sites, which were designed and run by government offices, humanitarian agencies or local religious groups, Ecoville was designed and run by a local university. Xavier University belongs to the prestigious Catholic family of Ateneo universities located in major Philippine cities. It donated five of the 17 hectares it owned in *barangay* Lumbia, in the outskirts of the city near the airport, for transitional and permanent resettlement housing for 550 Sendong survivor families (about 2700 individuals). Instead of limiting its involvement to financial and in-kind contributions, or to providing trained volunteers, as other Philippine universities have done in response to other disasters, the University spearheaded the planning and implementation of its site. The university had lofty goals: ‘Xavier Ecoville is envisioned to become an ecologically-friendly, self-reliant, God-centered community, designed as a model community in the Philippines and globally’ (Zech et al., 2018). Ecoville thus offers a (relocation house) window into answering Deneulin and Zampini-Davies (2017: 111)’s question ‘how do development [and post-disaster] actors, religious *and* secular, apply their beliefs and values to development [and relocation housing] programs, and how does the local context influence the application of these beliefs and values?’ The university staked its reputation on Ecoville’s success.

Officially, no prospective resident was excluded based on religion. As a key informant explained, the site ‘is very open to other religious beliefs. Like, it could not be one of the criteria for selection, or whatnot. Everybody is welcome in Xavier Ecoville. We make it a point that this is understood.’ There was even a debate on whether to build a chapel on site ‘because we [Catholics] would not want to dominate the entire community.’ Ultimately, the chapel was built, publicly cementing Ecoville’s Christian identity.

Xavier University commissioned Gawad Kalinga, a self-described Christian anti-poverty and nation-building movement that carries out social engineering projects (Gawad Kalinga, 2014), to implement a mandatory values formation program for prospective Ecoville residents. The Philippine-based NGO has extensive experience in building relocation housing and providing social assistance to survivors throughout the archipelago. The Ecoville program aimed to bring about a change in values, attitudes and behaviours among participants, which, in turn, would foster a cohesive community. All adult residents were required to complete an 8 day training before moving into a relocation house; they had to acquiesce to particular religious requirements portrayed as universal values. The training was expected to contribute to long-term success; once residents had assimilated the appropriate values – which they purportedly lacked pre-Sendong, it was assumed the residents would safeguard the proper social functioning of the relocation site.

The program content was adapted from a national Couples for Christ training program by local Gawad Kalinga staff to fulfil the perceived needs of Sendong survivors. The first part, called ‘Building community to end poverty,’ had three modules: (1) Called to care and share, (2) Building the community of our dreams, and (3) Together we can end poverty. The second part, called ‘Living a life of caring and sharing’, had four modules: (4) Loving the least, the last, the lost, (5) Loving without fear, (6) Loving is believing, and (7) Loving is life. Despite their clear identification as Christian values, Xavier University officials and Gawad Kalinga partners insisted the values were shared by people from all faiths.

We start with [becoming aware of your] feelings [and] valuing your neighbours, your community. And then values like service. [Then] you would have to address the needs of the poor, the excluded, and the lonely. Things like those. Those are very Christian values but of course, Muslim families are very much welcome. But these are [the] general kind of values that we think are important in building a community.

Christian values became barriers to entry. The values training, coupled with the absence of other trainings or activities to eliminate religious prejudices, reinforced pre-existing divisions and mobilities along religious lines. In the relocation sites, religious barriers were thus part of the institutional design.

Despite the absence of overt criteria requiring Christian affiliation, Ecoville’s mandatory values training all-but-ensured that survivors whose beliefs, as non-Christians, atheists or agnostics, did not align with Christian values did not become Ecoville residents. The requirements operated as a ‘governmobility’ whereby ‘spatial designs, physical infrastructures, and symbolic impediments creat[ed] divergent pathways, differential access, and control architectures for partial connectivity and bypassing’ (Sheller, 2016a: 16) created uneven mobilities for Christian and Muslim survivors. While the Muslim survivors themselves self-excluded, religious discrimination repelled them from Ecoville; in other words, the conditions were constructed such that the ‘natural’ choice of Muslim survivors would be to avoid Ecoville.

To recap, although observance of gender-based religious obligations yielded the same outcome for men and women, the impetus for the post-disaster mobilities of each group differed. Religious norms about veiling and the need for truly *private* private spaces dissuaded Muslim women from accessing evacuation camps, while religious norms about daily prayer inside of a mosque and the need to go to particular public spaces dissuaded Muslim men from accessing other official post-disaster spaces. Being able to practice one’s religion post-disaster was a salient aspect of post-disaster recovery. As O’Connell et al. (2017: 343) argue, based on their survey of nearly 2000 survivors of Typhoon Haiyan on Bantayan Island, Philippines, ‘a survivor’s ability to practice their faith, in a manner that takes the recent trauma into consideration, is important in helping to promote positive religious responses and to support emotional and psychological recovery.’ It is thus unsurprising that the *in*ability to continue the everyday socio-spatial experiences of religion shaped post-disaster mobilities in CDO, effectively turning Muslim survivors away from the official post-disaster sleeping spaces. Simply put, discrimination on the basis of religion deterred Muslim survivors from accessing official post-disaster spaces. Instead, Muslim survivors overwhelmingly utilized alternative mobilities, demonstrating a gendered post-disaster agency that required survivors to eschew official post-disaster spaces.

### Eschewing official post-disaster spaces

Religion pervades everyday post-disaster spaces. Religion matters to post-disaster mobilities, especially to exclusions from official post-disaster spaces. Religion turns these spaces into sites of resistance to mainstream disaster discourse (Aijazi and Panjwani, 2015). In CDO, a layered, multi-scalar exclusion was produced through the institutional design of evacuation camps, transitional sites and permanent relocation sites, and the self-exclusion by Muslim survivors themselves.

The first layer was self-exclusion, spurred by individual interpretations of religious beliefs and cultural practices. For reasons discussed in the preceding sections, Muslim survivors overwhelmingly rejected official post-disaster spaces, which in turn, limited their territorial mobility in the city. Self-exclusion resulted in a mostly immobile, rather than a trapped population. Affected survivors chose, to some degree, immobility.

That survivors prioritized religious needs and a sense of normalcy underscores the importance of what Aijazi and Panjwani (2015) describe as the purdah wall in disaster recovery. Pashtun survivors of the 2010 Pakistan Monsoon Floods prioritized rebuilding the purdah wall, even in temporary shelters. The purdah wall was deemed critical for rebuilding, especially for Muslim women’s everyday lives:

The ‘purdah wall’ is a socially sanctioned physical wall that surrounds a home or a collection of housing units; it is therefore a concrete manifestation of ‘purdah’. The walls allow women a safe space outside of the housing unit where they can freely move and interact with extended family without worrying about personal safety, gender segregation, and public notions of modesty. Essentially these walls extend the physical space available for women household members in the village setting. The purdah walls are therefore an extension of the social world available for women and help maintain the culturally mediated separation of the public (village life) and the private (womenfolk). (Aijazi and Panjwani, 2015: 39–40)

Despite its significance to survivors, the organizations working on shelter refused any assistance for constructing purdah walls.

A similar situation transpired in CDO, where disaster management actors overlooked the potential salience of the purdah wall and other gendered religious accommodations. Pre-existing conditions relegating the city’s Muslim population outside spaces of power persisted and were amplified post-disaster. The usual separation of spaces and activities along religious lines prevailed; for example, no Muslim representatives participated in the initial disaster response meetings held among key government, international humanitarian organization, university and religious actors. At the critical design stage, officials neither visited the houses of affected Muslims, nor spoke with them. If they had, officials may have noticed purdah walls, and heard requests for truly private spaces from Muslim women and proximity to mosques from Muslim men. But they did not. Unsurprisingly, official post-disaster spaces did not accommodate for the gendered socio-spatial practices of Muslim survivors. The resulting difficulty of incorporating religious beliefs into gendered everyday practices in official post-disaster spaces repelled Muslim survivors.

Muslim survivors’ deliberate self-exclusion on the basis on religion can thus be read as a demonstration of agency. Agency was exercised as the rejection of official evacuation camps, transitional housing sites and permanent housing sites in favour of utilizing resources from personal networks or clear religious spaces. Indeed, survivors’ initiatives to rebuild their lives and communities post-disaster can be difficult to initiate and sustain when post-disaster efforts are led by state, humanitarian and other non-governmental agencies (Santiago et al., 2018). Their agency is a resistance to humanitarian organization and government discourses and norms that treat people as undifferentiated survivor subjects (see Cupples, 2007; Hyndman, 1998, 2008, on the undifferentiated survivor subject). As in other disasters, for CDO’s Muslim survivors of Typhoon Sendong, religious discourses and gendered material practices derived from their interpretation emerged as the basis for contesting the undifferentiated survivor subject narrative. In CDO, immobility, and the rejection of official post-disaster spaces, were political acts. Such acts help ‘to redefine the political to include micro spaces of subversion within disaster relief: communities resist grand humanitarian narratives and poor program design by communicating in a language that feigns compliance with relief actors while preserving their dignity’ (Aijazi and Panjwani, 2015: 45).

Exclusion by institutional design was a second layer resulting both in Muslim survivors’ near territorial exclusion from official post-disaster spaces, and in Muslim survivors’ gendered everyday experiences of discrimination. The design of evacuation camps, transitional housing sites and permanent relocation settlements, in particular the absence of a purdah wall (Figure 1) and the geographic siting away from mosques, impeded specific gendered socio-spatial religious practices. Although government and humanitarian officials did not intend to erect religious barriers through camp and housing design, the same cannot be said for other exclusionary measures. For example, Muslim survivors were denied disaster relief, and even existence as legitimate disaster survivors. The state was complicit in such discrimination. To access survivor benefits, such as relief goods or free housing in a relocation site, individuals must be on an official survivor list. Not being on a list, or not being on a list that counts, rendered the survivor undocumented and illegitimate. Official post-disaster spaces were also linked to one dominant religion, as illustrated by Xavier Ecoville’s (Christian) values formation and God-centered aim. Discrimination with a religious basis thus operated as a governmobility, ‘disciplining … mobile subjects through affective experiences of moving with more or less friction, noise, danger, fear or turbulence’ (Sheller, 2016a: 16). CDO’s governmobilities spurred the conditions for uneven survivor mobilities and mobile agency as the most desirable option among Muslim survivors.

CDO’s government, (Christian) religious, and other officials noted the absence of Muslim survivors in official post-disaster spaces. Their explanations for this absence were generally framed as ‘it’s their culture,’ ‘they have lots of relatives,’ or ‘they go to the mosque.’ These problematic explanations sought to justify why official post-disaster efforts neglected the gendered needs and preferences particular to a religious minority. The explanations do not demonstrate a nuanced understanding religious identities and how embodied interpretations of these identities were mapped onto the everyday use of space and interactions with others. There was no consideration, for example, of the need for a purdah wall in the evacuation camp, or proximity to a mosque in the relocation site, or the outcomes of requiring values training based on Christian values. Without such considerations, key post-disaster actors facilitated particular post-disaster pathways for Sendong survivors, in part based on local stereotypes that further marginalized Muslim residents, and not on assessments of their actual needs and preferences.

## Conclusion

This article opened with a cheeky proposition – that natural hazards don’t care who you worship, but official post-disaster spaces do. It then proceeded to expound upon this assertion, using evidence from the post-disaster mobilities of Muslim survivors of Typhoon Sendong in CDO, Philippines. Discrimination along the lines of religion operated as what Sheller (2016a) describes as governmobility, ultimately deterring Muslim survivors from accessing evacuation camps, transitional housing sites and resettlement housing sites. Their exclusion from official post-disaster spaces result from gendered prejudices, preferences and practicalities, and from the socio-spatial design of official post-disaster spaces, or what Schewel (2020) categorizes as ‘retain’ and ‘repel’ factors, respectively. Cultural barriers and prejudices with a gendered religious basis thwarted motilities aimed at accessing official post-disaster spaces. These barriers were reinforced by the institutional design that discounted gendered religious requirements and preferences. These negative barriers dovetailed with a preference to draw upon family, friend and Islamic-centered networks. Together, these barriers and preferences reinforced each other in shaping Muslim survivors’ post-disaster mobilities. These mechanisms of exclusion produced a layered, multi-scalar exclusion. Cognisant of this discrimination, Muslim survivors exercised agency through various practices of moving and staying at various spatial and temporal scales.

These findings have import beyond the specific CDO case. They demonstrate how religion can operate in complex ways to produce uneven and discriminatory access to post-disaster resources. They raise questions about access to political and social justice, and especially disaster justice (Lukasiewicz, 2020). With environmental and disaster mobilities on an upward slope, there will be increasing demand for safe post-disaster refuges. Recognising the salience of religion to everyday gendered socio-spatial practices is thus critical in creating truly inclusive humanitarian spaces.

## Notes

1. According to Lukasiewicz (2020: 12–13), the four defining components of disaster justice are: (1) its positioning at the nexus of environmental, social and climate justice; (2) its relevance to the entire disaster management cycle and the construction and impacts of vulnerability; (3) its focus on distributive and procedural dimensions of justice; and (4) its attention to ecological justice, the needs of nonhumans during disasters, and the power of ecosystem based adaptation to climate change.

2. A *barangay* is a small administrative unit in the Philippines.

3. Various types of motorized and non-motorized vehicles provide transportation along set routes, and can also be used privately “for hire”. Some examples include the *motorela*, a small motorized scooter with an attached cab, the *jeepney*, a converted army van, the *multicab*, a small minibus, the *sikad*, a scooter, and the *pedicab*, a bicycle or tricycle with an attached cab.

4. A *malong* is a traditional woven tube skirt used by Muslim Filipino men and women in southern Mindanao and the Sulu archipelago. Today, it is widely used by Filipinos of all faiths throughout the archipelago as a skirt, a blanket in frigid Air Con buses, a hammock strung up in houses and *jeepneys* for sleeping children, a bed sheet, a prayer mat, and for many other uses. The *malongs* sold at the market are batik-style, which means they are constructed from machine-made printed cotton and much cheaper than traditional handwoven ones.

5. The regional office of the National Commission on Muslim Filipinos (NCMF-X) was the only notable exception, yet it too was marginalized by and isolated from other regional government offices. The office was situated, somewhat ironically, in *barangay* Nazareth, near the Nazareth mosque. Its isolation was due less to the physical distance separating it from other government offices, which were dispersed throughout the city, than to the minimal communication and coordination it had with other government offices except with other NCMF offices. This divide was unmasked in the relief and reconstruction efforts after Sendong. The appearance of the NCMF-X office also contrasted sharply with all the other municipal, provincial and regional government offices I visited. For example, it was very plain, and had minimal furniture, few computers and no air conditioning units.

6. *Amakan* is a type of locally available bamboo (*Bambusa* spp.) woven into large sheets and used in construction. As a lightweight and inexpensive building material, it is frequently used as walls or partitions by the urban poor in informal settlements and by middle class Cagayaños in their rural retreat houses. Several NGOs, notably the Catholic Relief Services (CRS), partnered with regional government agencies in issuing *amakan* sheets to Sendong survivors, which were used as walls in their temporary houses. When the survivor was transferred into a relocation house, they could keep or sell the *amakan* sheets.

7. *Nipa* is a type of palm tree (*Nypa fruticans*) found throughout the archipelago. Its fronds are frequently used to make the thatch roofs of the quintessential Philippine *nipa* hut. It is ideally suited for a tropical climate. If damaged by typhoons, floods or earthquakes, it can be easily repaired or rebuilt.

8. A bunkhouse is a quick construction housing unit; it is easy to construct and easy to dismantle. Its main advantage is the speed of construction. It is light construction and can be built easily with minimal engineering.
